# Multi-Process Action Control in Physical Activity: A Primer

**DOI:** 10.3389/fpsyg.2021.797484

**Published:** 2021-12-15

**Authors:** Ryan E. Rhodes

**Affiliations:** Behavioural Medicine Laboratory, School of Exercise Science, Physical and Health Education, University of Victoria, Victoria, BC, Canada

**Keywords:** exercise, attitude, intention-behavior gap, habit (automatism), identity, perceived behavioral control

## Abstract

The gap between the decision to engage in physical activity and subsequent behavioral enactment is considerable for many. Action control theories focus on this discordance in an attempt to improve the translation of intention into behavior. The purpose of this mini-review was to overview one of these approaches, the multi-process action control (M-PAC) framework, which has evolved from a collection of previous works. The main concepts and operational structure of M-PAC was overviewed followed by applications of the framework in physical activity, and concluded with unanswered questions, limitations, and possibilities for future research. In M-PAC, it is suggested that three layered processes (reflective, regulatory, reflexive) build upon each other from the formation of an intention to a sustained profile of physical activity action control. Intention-behavior discordance is because of strategic challenges in goal pursuit (differences in outcome vs. behavioral goals; balancing multiple behavioral goals) and automatic tendencies (approach-avoidance, conservation of energy expenditure). Regulatory processes (prospective and reactive tactics) are employed to hold the relationship between reflective processes and behavior concordant by countering these strategic challenges and automatic tendencies until the development of reflexive processes (habit, identity) begin to co-determine action control. Results from 29 observational and preliminary experimental studies generally support the proposed M-PAC framework. Future research is needed to explore the temporal dynamic between reflexive and regulatory constructs, and implement M-PAC interventions in different forms (e.g., mobile health), and at different levels of scale (clinical, group, population).

## Introduction

While most chronic health conditions have complex environmental, genetic, and behavioral etiologies (Kujala, 2011; Mulle and Vaccarino, 2013), adhering to regular health behaviors such as physical activity (PA) is critical to health promotion (Physical Activity Guidelines Advisory Committee, 2018; Chaput et al., 2020). A focus on understanding both initiation and long-term participation in regular PA is essential because participation rates at recommended levels are low (Guthold et al., 2018, 2019) with particularly steep declines among early PA initiates (e.g., Zuckerman, 2020). Understanding of the key antecedents of the PA enactment process using a theoretical foundation designed for behavior change intervention is desirable (Michie et al., 2014).

To this end, many theoretical approaches to understanding PA have been explored for over half a century (Rhodes et al., 2019b). The purpose of this mini-review is to overview the multi-process action control (M-PAC) framework and its potential utility in designing PA interventions as well as explaining certain PA behaviors. M-PAC has evolved within a collection of previous works, each with a focus on different aspects of the approach (Rhodes and de Bruijn, 2013b; Rhodes and Yao, 2015; Rhodes, 2017; Rhodes et al., 2021). This review is meant to act as a primer, linking to this prior work. The key aims of the review are to overview (a) the context for M-PAC in PA promotion, including its main concepts, and operational structure, and (b) highlight some applications of the framework. This overview concludes with unanswered questions, limitations, and possibilities for future research.

## Application of M-PAC

The approach taken in M-PAC extends from the social cognitive tradition of models in exercise and health psychology (Conner and Norman, 2015). Theories from this tradition each have unique constructs and formulations (e.g., social cognitive theory, theory of planned behavior, protection motivation theory), yet almost all suggest that expectations of the outcomes from behavioral action and the perception of one’s capability to perform PA determine the formation of a conscious goal or intention to perform a behavior, which is in-turn considered the determinant of subsequent PA (Rebar and Rhodes, 2020). M-PAC builds upon this tradition of goal-based behavioral performance with its focus on translating intentions into action, also known as action control (Kuhl, 1984). The importance of this approach is illustrated in many theories (see Heckhausen, 2007; Rhodes and Yao, 2015; Inzlicht et al., 2021 for overviews) and founded on long-standing evidence that most people have the intention to engage in PA, yet many people with these intentions do not follow-through, known now as the “intention-behavior gap” (Sheeran and Webb, 2016). Of particular importance, dichotomized explorations of the PA intention-behavior relationship show asymmetry, where nearly all non-intenders are congruent with their subsequent inaction, whereas approximately 50% of those with PA intentions engage in the intended PA (Rhodes and de Bruijn, 2013a). This clearly shows that intention is a necessary condition of such PA behavior for nearly all, but not sufficient to account for the actions of many.

The social cognitive foundation of M-PAC also highlights where it is likely best suited in PA interventions. Specifically, M-PAC is applicable to intention-based PAs such as exercise or other planned and purposeful activities. This represents a considerable number of PA promotion contexts (e.g., recreation programs, commuting, and all personal change decisions), yet spontaneous PA (unplanned activities), and PA performed as a more incidental means of achieving other directives (e.g., locomotion for other outcomes) are behaviors unlikely to benefit from an M-PAC approach. In addition, the social cognitive foundation of M-PAC also means that PA is conceived as a possible volitional behavior among other behavioral options. The M-PAC approach is unlikely to guide PA interventions where there is limited autonomy of choice (e.g., physical education classes, work policy) or in situations where the initial intention to engage in PA is not even viable (e.g., from large social inequities).

## Description and Function of M-PAC

**Figure 1:**
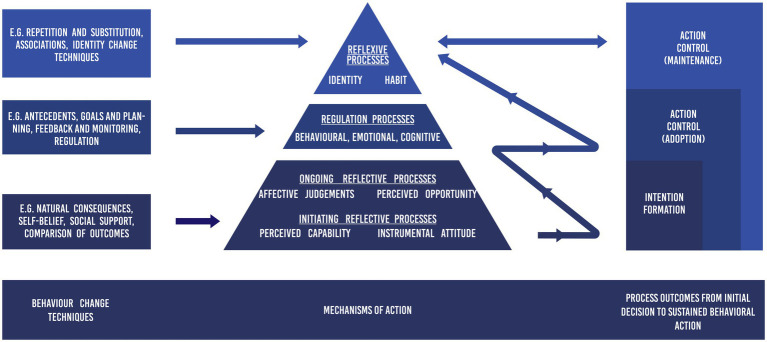
Multi-process action control schematic.

Multi-process action control is designed as a high-level meta-construction of PA behavior change from an initial decision to sustained behavioral patterns, based on several streams of past research and theorizing (see Rhodes, 2017 for a review). It features a schematic that can be explored through tests of falsifiability (Rhodes, 2017; Rhodes et al., 2021), yet the M-PAC was designed for PA intervention with practitioners in mind. Thus, the structure of the framework has a focus on the lateral relationship between behavior change techniques, their proposed theoretical mediators (or mechanisms of action) and resulting outcomes, more than the vertical interconnections between psychological constructs (See Figure 1).

Overall, M-PAC is a layered approach to behavior change that involves three connected, yet progressive processes (see mechanisms of action pyramid, Figure 1) that subsequently co-determine a sustained PA pattern of action control (see right aligned outcomes, Figure 1). It is proposed that these processes can be modified by specific external behavior change techniques (see left aligned boxes, Figure 1), but also naturally build-upon and co-determine each other through experiences with the resulting behavioral outcomes (see the snaking arrow, Figure 1). *Reflective processes* are foundational, and represent the consciously deliberated expectations of performing PA. Reflective processes are based in the social cognitive tradition, suggesting that expectations are largely predicated on judgments from past experiences, social comparisons, and information (Bandura, 1998; Fishbein and Ajzen, 2010). Commensurate with this social cognitive tradition, specific behavior change techniques (e.g., natural consequences, comparison of outcomes; see Rhodes, 2017 for a detailed list) impact reflective processes through intervention. The proposed mechanisms of action are *instrumental attitude* (evaluation of the expected benefits from performing PA), *affective judgments/affective attitude* (evaluation of the expected pleasure from performing PA), *perceived capability* (evaluation of one’s ability to perform PA, circumscribed from motivation), and *perceived opportunity* (evaluation of the perceived social and environmental circumstances to perform PA, circumscribed from motivation). When these expectations are strong and positive, they culminate with the formation of PA intention, which is defined as the decision to enact regular PA (Rhodes and Rebar, 2017). In the M-PAC framework, however, reflective processes of perceived opportunity and affective judgments are also posited to predict the translation of an intention into behavior, to the extent that they represent a proxy for the affective and logistical factors that challenge one’s competing daily decisions (known as ongoing reflective processes; Rhodes, 2017). By contrast, instrumental attitude and perceived capability are not considered antecedents of the translation of an intention because they are expected to vary less from day to day and situation to situation (known as initiating reflective processes; Rhodes, 2017). This is a distinction made in M-PAC when compared to most other action control approaches that position intention as the final motivational outcome in the behavioral process (Rhodes and Yao, 2015). Instead, it is contended that follow-through of initial intentions will be partially dependent on strong affective judgment and perceptions of opportunity (Rhodes, 2017).

Still, while reflective processes may affect some aspects of action control, the dominant determinant when beginning regular PA is marked by the enactment of *regulatory processes (*see Figure 1 middle of the pyramid*). Regulatory processes* represent the behavioral, cognitive, and affective regulation tactics that are employed to translate a formulated intention into PA. These can be prospective (e.g., planning, monitoring, restructuring the built and physical environment) or reactive (e.g., emotion regulation, attentional focus) in their implementation and have been described and applied extensively in models of goal pursuit (e.g., Carver and Scheier, 1982; Bandura, 1986; Bagozzi, 1992; Karoly, 1992; Gollwitzer, 1999; Locke and Latham, 2006; Schwarzer, 2008; Mann et al., 2013; Duckworth et al., 2016). Engaging in regulatory processes may be a seamless consequence of intention formation (i.e., spontaneous behavioral regulation; see snaking arrow in Figure 1), or through the result of outside intervention (Allan et al., 2013; Carraro and Gaudreau, 2013; see left aligned behavior change techniques in Figure 1). Their function in M-PAC can take multiple paths (see Rhodes et al., 2021), yet their main role is essentially to act like the metaphorical glue between reflectively processed intention and PA, long enough for reflexive processes to begin to co-determine action control (see right aligned outcomes, Figure 1). While simple behaviors may be co-determined by a dual (reflective and reflexive) process model configuration (Deutsch and Strack, 2006), Rhodes et al. (2021) contend that complex behaviors such as PA face ongoing strategic challenges and automatic tendencies that thwart intention-behavior correspondence, and thus necessitate regulatory processes as a bridge to action control.

Strategic challenges are those that prohibit the sustained execution of intention-behavior relations prospectively, often long before PA has been attempted, such as outcome vs. behavioral goal confusion and the failure to consider multiple goals (Rhodes et al., 2021). For example, the intention to engage in PA is often linked to the desired long-term outcomes of PA, such as weight control, fitness, and reducing the chances of chronic disease and rarely linked to the performance of PA itself (Symons Downs and Hausenblas, 2005). This is an enormous strategic error in intention formation because of the time and effort needed to sustain behaviors such as PA for such long-term outcomes (Rhodes and Nigg, 2011). Thus, regulatory processes that help focus on the behavioral experience itself (Segar and Richardson, 2014), and not distal outcomes, have well established efficacy (Locke and Latham, 2006). Another key strategic challenge is the failure to consider multiple goals (Little, 1983), which is likely to under-estimate the resources to perform other behaviors (Rachlin et al., 1980; Orehek and Vazeou-Nieuwenhuis, 2013). Indeed, juggling multiple goals is one of the prime reasons why new intentions are often abandoned (Sheeran and Webb, 2016). PA goals that compete for time and resources (i.e., goal conflict) increase the intention-behavior gap, compared to goals that can be mutually achieved with the same actions (goal facilitation; Rhodes et al., 2016b). Regulatory processes that assist in helping with the prioritizing of one intention over another (e.g., planning), thus becomes an important strategic approach to successful behavioral performance (Freund and Baltes, 2002; Conner et al., 2016).

Rhodes et al. (2021) suggest that action control can also be disrupted by automatic tendencies and these also require regulatory processes to counteract their proximal impact on the intention-behavior gap. For example, people have a basic underlying tendency to approach experiences that are pleasant and avoid experiences that are unpleasant (Cabanac, 1992). Several research teams have theorized and provided evidence that unpleasant affective experiences with PA may impact future behavior below reflective awareness, or at least preceding cognitive reflection (Rhodes and Kates, 2015; Conroy and Berry, 2017; Brand and Ekkekakis, 2018; Stevens et al., 2020). Regulatory processes that attempt to counteract this effect are hypothesized as critical to translate positive PA intentions into behavior (Rhodes and Gray, 2018). Relatedly, research stemming from evolutionary biology has supported a basic human tendency to minimize metabolic costs which stems from an evolutionary survival necessity (Lieberman, 2015; Cheval and Boisgontier, 2021). Thus, fulfilling PA intentions that do not involve necessity, such as exercise, are likely to be met with an underlying avoidance tendency, because it represents unnecessary energetic cost (Cheval et al., 2016). Regulatory processes are theorized to help counteract this tendency (e.g., planning PA when one has the most energy; adding PA to more meaningful tasks) and aid in fulfilling PA intentions (Rhodes et al., 2021).

Finally, *reflexive processes* in M-PAC are those constructs that develop as a consequence of repeated action control over time (Rhodes, 2017). M-PAC includes habit (learned cue-behavior associations) and identity (role self-categorization) as the two key reflexive constructs (see top of the pyramid; Figure 1); their interrelationship and proposed antecedents are detailed in Rhodes et al. (2021). Like all layers of the M-PAC pyramid, however, habit and identity are proposed to arise as a natural consequence of repeated successful behavioral outcomes (see snaking arrow, Figure 1) and can be intervened upon through specific external behavior change techniques (e.g., associations, repetition, identity formation strategies; see left aligned box in Figure 1). Action control habits are based on the premise that complex behaviors, such as PA, do not comprise of an all-or-nothing habit response, but instead assist in automating certain sub-components of a larger behavioral sequence (Gardner et al., 2016; Phillips and Gardner, 2016; Rhodes and Rebar, 2018; Gardner et al., 2020). Habit in M-PAC assists primarily as a form of selection bias toward intended action (see Rhodes et al., 2021 for a detailed review). Identity is thought to fulfil a similar role of selection bias in action control. Essentially, those with a PA identity are theorized to be more attuned to seize opportunities to be active and thus fulfil their intentions (see Rhodes et al., 2016a for a review). However, identity may also playback into regulatory processes by bolstering the motivation to use tactics to fulfil PA intentions (Rhodes et al., 2021). Overall, the formation of habit and identity are proposed to engender sustained action control through greater enactment efficiency (see right aligned categories in Figure 1), in part by replacing the use of regulatory processes (Rhodes and Sui, 2021) and lessening the cognitive demands of reflective processes (Caldwell et al., 2018).

Taken together, the M-PAC constructs have a causal structure that progresses from intention formation, to initiated action control and onto sustained action control, but each process has reciprocal and reinforcing relationships (see Rhodes, 2017; Rhodes et al., 2021). Thus, while the M-PAC schematic represents an ordered acquisition of reflective, regulatory, and reflexive processes that build upon each other over time, each is expected to have some mediated feedback onto behavior and intention along with their own independent effect. The tri-partite approach to promoting reflective, regulatory, and reflexive mechanisms of action to enact intention-behavior correspondence is a core feature of M-PAC (Rhodes, 2017).

## Application of M-PAC

Because M-PAC is a meta-construction of multiple lines of behavioral research in PA, there is already considerable evidence for the importance of reflective, regulatory, and reflexive processes in both behavioral prediction and behavior change. For example, the importance of a distinction between affective judgments and instrumental attitude in understanding and intervening upon PA has been established in reviews and meta-analyses (e.g., Rhodes et al., 2009; Nasuti and Rhodes, 2013; Connell Bohlen et al., 2021). Reviews also show the specific effect of affective judgments on action control (e.g., Rhodes and de Bruijn, 2013b; McEachan et al., 2016; Rhodes et al., 2019a). Similar reviews are present for the key roles of perceived capability (e.g., Williams and French, 2011; Williams and Rhodes, 2014; Young et al., 2014; McEachan et al., 2016), opportunity (e.g., Michie et al., 2011; Rhodes, 2017), regulatory processes (e.g., Gollwitzer and Sheeran, 2006; Michie et al., 2009; Bélanger-Gravel et al., 2013; Carraro and Gaudreau, 2013; Kwasnicka et al., 2013; McEwan et al., 2016), habit (e.g., Gardner et al., 2011; Rebar et al., 2016), and identity (Rhodes et al., 2016a). Thus, the independent contributions of the constructs in M-PAC have strong empirical support.

The multivariate tests of M-PAC constructs was recently reviewed in Rhodes et al. (2021). The authors reported on 26 independent studies that had used either a full M-PAC approach or a variant (>75% of the variables present) and three additional studies have been published since that time (see Supplementary Table S1 for reference list). Eleven studies specifically tested the tenet that PA action control is a consequence of reflective, regulatory, and reflexive processes. Seven of these tests supported significant independent contributions of each process, with the remaining four tests showing reflexive and regulatory constructs as key determinants. Specific construct-level tests of M-PAC variables can be found in Table 1. For reflective processes, instrumental attitude was a predictor of intention-PA translation in 2 of 18 tests, and affective judgment was a significant positive predictor in 12 of 16 tests Perceived opportunity and capability were predictors in 3/5 and 0/5 tests, respectively, yet most studies have employed an amalgam measure of perceived behavioral and this was significant in 9 of 15 tests. Regulatory processes was a significant predictor of action control in 13 of 16 studies. Two studies specifically showed that regulation processes predicted action control for participants in adoption (defined in these studies as participating in PA < 6 months) more than maintenance (participating in PA > 6 months). Finally, both identity (10 of 10 tests) and habit (11 of 12 tests) were reliable predictors of action control, yet no study distinguished these relationships by adoption and maintenance. Of the seven experimental applications, six showed significant evidence of behavior change across time and all studies showed changes from baseline in M-PAC target constructs (see Rhodes et al., 2021).

**Table 1:** M-PAC variables as predictors of the intention-physical activity behavior gap using observational designs.

Construct	Number of Studies with reported Effect Sizes	Median Effect Size *d* in Bivariate Tests	Range	Significant Associations in Multivariate Tests
Affective Judgments	14	0.47	0.13–0.77	12/16
Instrumental Attitude	16	0.21	−0.10–0.58	2/18
Perceived control	13	0.40	0.05–1.00	9/15
Capability	4	0.42	0.05–0.60	0/5
Opportunity	4	0.38	0.13–0.63	3/5
Behavioral Regulation	12	0.39	−0.06–0.78	13/16
Habit	9	0.66	0.32–0.93	11/12
Identity	5	0.73	0.66–0.89	10/10

*Effect size d is estimated across studies where possible and thus the number of studies differs from the multivariate significance tests*.

## Conclusion and Future Directions

The gap between the decision to engage in PA and subsequent behavioral enactment is considerable for many. M-PAC is a theoretical framework created to promote greater success in translating positive intentions into behavior; and this primer paper overviewed the main conceptions of the approach and its core constructs. While M-PAC may be a helpful approach to use for the promotion of some PAs, there are certainly areas for future research and development. For example, distinctions between maintenance and initiation in M-PAC is denoted as a dynamic between the growing predictive capabilities of reflexive constructs that partially supplant regulatory and reflective processes, yet this has seen scant attention. Future research employing dynamic models with intensive longitudinal data (Ruissen et al., 2021) is required to explore this tenet. In terms of experimental validation, the constructs within M-PAC are also at different levels of validity testing. Overall, there is preliminary evidence that M-PAC constructs are changeable and that these changes may result in subsequent PA change, but there is limited evidence about the relative effectiveness of targeting each of the reflective, regulatory, and reflexive layers and the precision of the behavior change techniques to accomplish this aim. There is also limited evidence on how to implement M-PAC interventions in different forms (e.g., mobile health, inter-personal, just-in-time interventions), and at different scales (clinical, group, population). Further, while many of the constructs in M-PAC have a large evidence base from parent domains of health and social psychology, the relationship between perceived capability and opportunity, and identity and habit need considerably more research attention. Indeed, the domain of reflexive processes in PA is still within its infancy with many different directions that are promising to create a more complex and informative understanding of PA (Rebar et al., 2016; Chevance et al., 2019; Stevens et al., 2020) that will need consideration in future variations of the M-PAC schematic.

## Author Contributions

The author confirms being the sole contributor of this work and has approved it for publication.

## Conflict of Interest

The author declares that the research was conducted in the absence of any commercial or financial relationships that could be construed as a potential conflict of interest.

The handling editor declared a past collaboration with the author.

## Publisher’s Note

All claims expressed in this article are solely those of the authors and do not necessarily represent those of their affiliated organizations, or those of the publisher, the editors and the reviewers. Any product that may be evaluated in this article, or claim that may be made by its manufacturer, is not guaranteed or endorsed by the publisher.
