# Supplementary material for: Multi-Process Action Control in Physical Activity: A Primer
Source: Front Psychol. 2021 Dec 15;12:797484. doi: 10.3389/fpsyg.2021.797484 (PMC8714894; doi:10.3389/fpsyg.2021.797484)
Supplement: Supplementary file 1 [file Table_1.docx]

Supplementary Table 1

M-PAC References of Empirical Tests

Observational Designs

Bassett-Gunter, R.L., Tanna, S., Arbour-Nicitopoulos, K.A., Rhodes, R.E., & Leo, J. (2020). Understanding the role of parents in supporting physical activity among children and youth with disabilities: A behavior change theory perspective. European Journal of Adapted Physical Activity. 13(11), 1-16.

de Bruijn, G. J., de Groot, R., van den Putte, B., & Rhodes, R. E. (2009). Conscientiousness, extroversion, and action control: comparing moderate and vigorous physical activity. *Journal of Sport and Exercise Psychology, 31*, 724-742.

de Bruijn, G. J. (2011). Exercise habit strength, planning and the theory of planned behaviour: An action control approach. *Psychology of Sport and Exercise, 12*, 106-114.

de Bruijn, G. J., Verkooijen, K., de Vries, N. K., & van den Putte, B. (2012). Antecedents of self identity and consequences for action control: An application of the theory of planned behaviour in the exercise domain. *Psychology of Sport and Exercise, 13*, 771-778.

de Bruijn, G. J., & Van den Putte, B. (2012). Exercise promotion: An integration of exercise self-identity, beliefs, intention, and behaviour. *European Journal of Sport Science, 12*, 354-366.

Godin, G., Shephard, R. J., & Colantonio, A. (1986). The cognitive profile of those who intend to exercise but do not. *Public Health Reports, 101*, 521-526.

Grant, S., Lithopoulos, A., & Rhodes, R.E. (2021). Understanding action control of physical activity among mothers with young children. International Journal of Sport and Exercise Psychology, Online First, 1- 17.

Fiala, B., & Rhodes, R. E. (2014). Understanding exercise action control: Moderation of intensity on physical activity intentions and behavior in a Canadian university sample. In V. L. Rush (Ed.), *Planned Behavior: Theory, Applications and Perspectives* (pp. 73-94). New York: Nova Publications.

Kwan, M., Brown, D., Dutta, P., Haider, I., Cairney, J., & Rhodes, R.E. (in press). Application of the Multi-Process Action Control Model to predict physical activity during late adolescence. Journal of Sport & Exercise Psychology.

Rhodes, R.E., Beauchamp, M.R., Quinlan, A., Symons Downs, D., Warburton, D.E.R., & Blanchard, C.M. (2021). Predicting the physical activity of new parents who participated in a physical activity intervention. Social Science & Medicine, 284, 114221

Rhodes, R. E., & Lithopoulos, A. (in press). Understanding action control of resistance training among adults. *Psychology of Sport & Exercise*.

Rhodes, R. E., & Plotnikoff, R. C. (2006). Understanding action control: Predicting physical activity intention-behavior profiles across six months in a Canadian sample. *Health Psychology, 25*, 292-299.

Rhodes, R. E., Courneya, K. S., & Jones, L. W. (2003). Translating exercise intentions into behavior: Personality and social cognitive correlates. *Journal of Health Psychology, 8*, 447-458.

Rhodes, R. E., Plotnikoff, R. C., & Courneya, K. S. (2008). Predicting the physical activity intention-behaviour profiles of adopters and maintainers using three social cognition models. *Annals of Behavioral Medicine, 36*, 244-252.

Rhodes, R. E., de Bruijn, G. J., & Matheson, D. H. (2010). Habit in the physical activity domain: Integration with intention temporal stability and action control. *Journal of Sport and Exercise Psychology, 32*(1), 84-98.

Rhodes, R. E., Nasuti, G., & Fiala, B. (2012). Action control of exercise behaviour: Evaluation of social cognition, cross-behavioural regulation and automaticity. *Behavioral Medicine, 38*, 121-128.

Rhodes, R. E., Spence, J. C., Berry, T., Deshpande, S., Faulkner, G., Latimer-Cheung, A., . . . Tremblay, M. (2016). Understanding action control of parent support behavior for child physical activity. *Health Psychology, 35*, 131-140. doi: 10.1037/hea0000233

Rhodes, R. E., & Lim, C. (2016). Understanding Action Control of Daily Walking Behavior among Dog Owners: A Community Survey. *BMC Public Health, 16*, 1165-1174.

Rhodes, R. E., Berry, T., Faulkner, G., Latimer-Cheung, A. E., O’Reilly, N., Tremblay, M. S., . . . Spence, J. C. (2019). Application of the multi-process action control framework to understand parental support of child and youth physical activity, sleep, and screen time behaviors. *Applied Psychology: Health and Well-Being, 11*, 223-239.

Rhodes, R.E., Quinlan, A., Naylor, P.J., Warburton, D.E.R., & Blanchard, C.M. (2020). Predicting personal physical activity of parents during participation in a family intervention targeting their children. Journal of Behavioral Medicine, 43, 209-224.

Vallerand, J., Rhodes, R. E., Walker, G. J., & Courneya, K. S. (2016). Understanding strength exercise intentions and behavior in hematologic cancer survivors: An analysis of the intention-behavior gap. *Journal of Cancer Survivorship, 10*, 144-147.

Wienert, J., Kuhlmann, T., Storm, V., Reinwand, D., & Lippke, S. (2019). Latent user groups of an eHealth physical activity behaviour change intervention for people interested in reducing their cardiovascular risk. *Research in Sports Medicine, 27*, 34-49.

Experimental designs

Tanna, S., Arbour-Nicitopoulos, K., Rhodes, R. E., & Bassett-Gunter, R. (2017). A pilot study exploring the use of a telephone-assisted planning intervention to promote parental support for physical activity among children and youth with disabilities. *Psychology of Sport and Exercise, 32*, 25-33. doi: 10.1016/j.psychsport.2017.05.003

Kaushal, N., Rhodes, R. E., Spence, J., & Meldrum, J. (2017). Increasing physical activity through principles of habit formation in new gym members: A randomized-controlled trial. *Annals of Behavioral Medicine, 51*, 578-586.

Lim, C., Wharf Higgins, J., & Rhodes, R. E. (in press). Working out with F.I.D.O. (frequency, intensity, duration, & outcomes): A feasibility randomized controlled trial. *Human-Animal Interaction Bulletin*.

Rhodes, R. E., Naylor, P. J., & McKay, H. A. (2010). Pilot study of a family physical activity planning intervention among parents and their children. *Journal of Behavioral Medicine, 33*, 91-100.

Vallerand, J. R., Rhodes, R. E., Walker, G. J., & Courneya, K. S. (2018). Feasibility and preliminary efficacy of an exercise telephone counselling intervention for hematologic cancer survivors: A phase II randomized controlled trial. *Journal of Cancer Survivorship, 12*, 357-370.

Rhodes, R. E., Naylor, P. J., Blanchard, C., Quinlan, A., & Warburton, D. E. R. (2019). Family physical activity planning and child physical activity outcomes: A randomized trial. *American Journal of Preventive Medicine, 57*, 135-292.

Husband, C., Wharf Higgins, J., & Rhodes, R. E. (2019). A feasibility randomized trial of an identity-based physical activity intervention among university students. *Journal of Health Psychology and Behavioral Medicine, 7*, 128-146.
